# Parietal Lobe Reorganization and Widespread Functional Connectivity Integration in Upper-Limb Amputees: A rs-fMRI Study

**DOI:** 10.3389/fnins.2021.704079

**Published:** 2021-07-20

**Authors:** Bingbo Bao, Haifeng Wei, Pengbo Luo, Hongyi Zhu, Wencheng Hu, Yi Sun, Junjie Shen, Tianhao Zhu, Junqing Lin, Tengli Huang, Jing Li, Zhibin Wang, Yuehua Li, Xianyou Zheng

**Affiliations:** ^1^Department of Orthopedic Surgery, Shanghai Jiao Tong University Affiliated Sixth People’s Hospital, Shanghai, China; ^2^Institute of Diagnostic and Interventional Radiology, Shanghai Jiao Tong University Affiliated Sixth People’s Hospital, Shanghai, China

**Keywords:** amputation, body image disorder, phantom sensation, phantom pain, functional magnetic resonance imaging, functional connectivity, ALFF

## Abstract

The right parietal lobe plays an important role in body image, and disorders of body image emerge after lesions in the parietal lobe or with parietal lobe epilepsy. Body image disorder also often accompanies upper-limb amputation, in which the patient misperceives that their missing limb is still part of their body. Cortical reorganization is known to occur after upper-limb amputation, but it is not clear how widespread and to what degree functional connectivity (FC) is reorganized post-amputation, nor whether such changes might be related to misperceptions of body image. Twenty-four subjects who had a traumatically upper-limb amputees (ULAs) and 24 age-matched healthy controls (HCs) underwent resting-state functional magnetic resonance imaging (rs-fMRI) scans. Regions of interest (ROIs) in the right superior parietal gyrus (SPG_R) and right inferior parietal lobule (IPL_R) were defined using BrainNet Viewer. We calculated the amplitude of low-frequency fluctuations (ALFF) in ROIs and correlated the ROI mean amplitude of low-frequency fluctuations (mALFF) and mean scores on the phantom limb sensation (PLS) scale and beck depression index (BDI). We also calculated ROIs and whole-brain FC. Compared to the HC group, we observed significantly increased activation (mALFF) in ROIs of the ULA group. Moreover, correlation analyses revealed a significant positive correlation between ROI mALFF and scores on the PLS. There was a significant negative correlation between the SPG_R mALFF and BDI scores. Seed-based, whole-brain FC analysis revealed that FC in the ULA group significantly decreased in many brain regions across the entire brain. The right parietal lobe appears to be involved in some aspect of body awareness and depression in amputation patients. Upper-limb amputation results not only in reorganization in the local brain area formerly representing the missing limb, but also results in more widespread reorganization through FC changes in whole brain.

## Introduction

Limb amputation is an important health issue affecting the quality of life of untold numbers of people worldwide (Pomares et al., 2018). In the United States, for example, the number of limb amputees continues to increase, mostly due to increases in the number of traumatic injuries resulting from traffic accidents and natural disasters, and amputations related to diabetes and malignant tumors of limbs (Varma et al., 2014). This increasing trend can be seen worldwide, with 1.5 amputations being performed per 1000 people. Although amputation can save lives, the risk of many types of medical complications remains. Moreover, sensory disorders and psychological problems can be present (Armstrong et al., 2019).

Phantom limb pain (PLP), residual limb pain (RLP), and phantom limb sensation (PLS) are the most common clinical complications of amputations (Woodhouse, 2005; Kaur and Guan, 2018; Stover and Prahlow, 2020). Recent studies indicate that 60–80% of amputees experience phantom pain and approximately 80–100% experience phantom sensation (Jensen et al., 1983; Urits et al., 2019). Faced with such high incidences of complications, much basic research has focused on better understanding quality-of-life reducing complications, especially those involving PLP. Theories explaining PLP mainly involve peripheral, central, and supraspinal mechanisms (Flor, 2002; Flor et al., 2006). Despite various useful treatments for PLP (Erlenwein et al., 2021), it still cannot be completely resolved clinically and thus continues to seriously affect amputees’ quality of life.

Another consequence of upper-limb amputations is body image disorder. Body image disorder, or disturbance, refers to several different conditions in which a person’s body image mismatches reality; that is, the patient feels extreme anxiety and fear associated with an imagined or minor physical flaw, which significantly impedes normal, everyday functioning. Body image disorder comprises several different body disturbances recognized by the DSM-V, including body dysmorphic disorder and muscle dysmorphia, among others. Basic perceptual functions of patients are normal, but the existence of their own body parts, their spatial position, and the relationship between each part are distorted (Demirdel and Ülger, 2021). Amputees often suffer from body image disorders. For example, even though their limb is absent, they perceive that their limb still is present. This kind of disturbance in body image has been linked to various negative psychosocial outcomes, ones involving perceptual, affective, cognitive, evaluative, and behavioral disturbances (McDonald et al., 2014; Luza et al., 2020).

Many studies have investigated the reorganization of the nervous system after amputation, but usually from the perspective of understanding PLS. PLS may be the result of ongoing neuroplasticity (Mercier et al., 2006; Di Pino et al., 2009). Other studies have reported strong correlational relationships between PLS and the degree of cortical reorganization (Wheaton, 2017). A separate line of research on body image disorders shows that the right parietal cortex is prominently involved in the disorder. The right parietal cortex represents a higher-order convergence zone of somatosensory, visual, and vestibular input that is critical for sensorimotor integration (Wolpert et al., 1998). This integration of sensory information with motor intention and actions represents the core of a unified sense of the body in space (Tsakiris, 2010). We are unaware of any relevant research on whether brain remodeling in the parietal lobe after amputation might be related to body image disorders in upper-limb amputees (ULAs).

Resting-state functional magnetic resonance imaging (rs-fMRI) is a promising tool for analyzing brain function remodeling and functional connectivity (FC). rs-fMRI studies represent a significant approach for researching different diseases and disorders at the brain-network level (Smitha et al., 2017). rs-fMRI does not require participants to perform any complex sensorimotor task; it monitors intrinsic activity within the brain, in the absence of any sensory or cognitive stimulus. Resting-state FC analysis has been used to study network-level reorganization of FC following arm amputation, and has revealed reduced FC between neocortical areas associated with the missing hand and the sensorimotor network in amputees (Makin et al., 2015).

Since PLS are manifested in an incorporeal body part, they can be regarded as one type of body image disorder, one that is closely related to changes in the right parietal lobe (Sadibolova et al., 2019). Prompted by the above considerations, we predict that the right parietal lobe in ULAs will undergo changes in plasticity and FC integration, which might be detected with rs-fMRI and network analysis (Smitha et al., 2017). Therefore, the aim of the present study was to characterize right parietal lobe plasticity following upper-limb amputation and to determine its relationship to the phenomenology of PLS.

## Materials and Methods

### Participants

Characteristics of participating subjects are summarized in Table 1. Twenty-four individuals (19 male and 5 female) with acquired unilateral upper-limb amputation (mean age ± SD: 44.67 ± 8.33; 15 patients with amputations on the right side) were recruited through the department of orthopedic surgery of a large metropolitan tier 1 hospital in China between October 2020 and December 2020. Thirteen amputations occurred above the elbow and 11 occurred below the elbow. All the patients underwent amputation following a traumatic injury. Exclusion criteria were the following: (1) upper-limb amputation along with another part of the body; (2) history of neurological disease, diabetes, or previous neurotrauma; (3) presence of neurological or psychiatric disorders; (4) elapsed time between amputation and MRI scanning was <3 months; (5) history of psychotropic drug use or (6) MRI contraindication. Twenty-four limb-intact individuals matched for age (mean age, range in years); education; and sex served as healthy controls (HCs). These participants were recruited from the local community. All subjects were right-handed, as assessed by the Chinese version of the Edinburgh Handedness Inventory (Yang et al., 2018). Each participant was informed of the purpose and methods of the study, and each signed a written informed consent to participate. The study was approved by the ethics committee of our institution and performed according to international standards (World Medical Association., 2013).

**TABLE 1:** Demographic and clinical characteristics of participating upper-limb amputees.

**Subject no.**	**Age/sex**	**Side/level of amputation**	**Education (years)**	Time*	**RLP^†^**	**PLP^‡^**	**PLS^§^**	**BDI**	**BAI**
1	29/M	L/AEA	7	38	1	2	7	2	3
2	44/F	R/BEA	12	36	0	1	4	1	7
3	41/M	L/AEA	9	32	6	4	9	1	1
4	41/M	L/BEA	9	30	1	1	1	5	21
5	38/M	R/AEA	9	34	2	3	10	3	4
6	56/M	L/BEA	0	43	6	6	8	22	33
7	48/F	R/AEA	8	32	6	6	4	13	9
8	42/M	R/AEA	2	47	1	1	5	7	28
9	52/M	R/AEA	8	26	7	6	3	33	46
10	41/M	L/AEA	9	18	5	8	6	17	17
11	54/M	L/AEA	5	264	5	5	9	1	8
12	59/M	R/AEA	10	5	0	3	7	9	2
13	47/M	L/BEA	12	26	1	0	4	12	3
14	42/M	L/BEA	8	46	0	0	2	6	4
15	50/M	R/AEA	8	348	0	2	8	2	1
16	39/M	R/BEA	7	60	0	0	10	4	15
17	44/M	R/BEA	12	48	1	1	6	4	5
18	45/F	R/AEA	3	7	3	7	3	4	27
19	37/M	R/BEA	22	4	5	7	10	3	5
20	40/M	R/AEA	12	48	7	5	8	17	11
21	27/M	R/BEA	15	51	1	0	4	2	0
22	60/F	R/BEA	3	300	3	2	7	19	22
23	43/M	L/AEA	8	252	2	0	10	1	1
24	53/F	R/BEA	8	432	0	0	6	6	4

M, male; F, female; R, right; L, left; AEA, above the elbow; BEA, below the elbow; RLP, residual limb pain; PLP, phantom limb pain; PLS, phantom limb sensation; Chinese version of BDI, beck depression index, 0 = Not at All to 63 = severe; Chinese version of BAI, Beck anxiety inventory, 0 = Not at All to 63 = severe.

*Elapsed time in months from amputation.

*^†^Residual limb pain, 0 = none to 10 = worst imaginable at time of MRI.*

*^‡^Phantom limb pain, 0 = none to 10 = worst imaginable at time of MRI.*

*^$^Phantom Limb Sensations, 0 = none to 10 = worst imaginable at time of MRI.*

### Clinical Assessments

Clinical assessments were done before fMRI scanning. An adapted questionnaire for upper limb amputation was used to collect information about amputation-related variables. The adaptations assessed the level of amputation, side of amputation, elapsed time (months) since amputation, previous treatment approaches, and whether they were effective or not. We also assessed RLP; PLP; and the frequency, quality, and type of PLS. Pain was measured with a visual analog scale assessment tool, on which self-reported pain is scored on a scale from 0 (no pain) to 10 (worst imaginable pain). As a visual indicator of pain, this scale was also color-coded with a gradient of green (at 0) to red (at 10). Participants were asked to rate the presence and intensity of pain related to PLP. Similar scales were used to assess RLP, stump pain, and PLS, and non-painful sensations. Depression and anxiety, respectively, was evaluated using the Chinese versions of the Beck Depression Index (BDI) and Beck Anxiety Index (BAI).

### MRI Scanning and Image Acquisition

For image acquisition of functional and structural data, we used a Siemens 3.0-T MRI scanner (MAGNETOM Prisma; Siemens Healthcare GmbH, Erlangen, Germany) equipped with a 64-channel phased-array head coil. Rs-fMRI data were collected via simultaneous multi-slice MRI technology for a total of 240 volumes (288 s). The following parameters were used: repetition time (TR) = 1200 ms; echo time (TE) = 39 ms; flip angle = 52°; matrix = 88 × 88; field of view = 100 mm; slice thickness = 2.4 mm; 56 slices with a voxel size = 2.4 mm × 2.4 mm × 3.0 mm. During the resting-state scan, subjects were asked to relax with their eyes closed and not to think of anything in particular. In addition, for each participant we acquired high-resolution T1-weighted structural images using a magnetization-prepared rapid gradient echo (MPRAGE) pulse sequence. The following parameters were used: TR = 2300 ms; TE = 2.46 ms; flip angle = 8°; matrix = 256 × 256; thickness = 1.0 mm; 176 slices with a voxel size = 1 mm × 1 mm × 1 mm.

### Data Preprocessing

Preprocessing was performed using Statistical Parametric Mapping (SPM12^1^) implemented in MATLAB R2013b (MathWorks Inc., Natick, MA, United States). To allow the signal to reach equilibrium and the participants to adapt to the scanner noise, data collection began after 10 images were collected; thus, these first 10 scans of each subject were discarded. The remaining 230 images of each subject were co-registered to the individual anatomical data sets after the anterior commissure had been manually defined as the reference point. For all subjects, the translation or rotation parameters did not exceed ±2.5 mm or ±2.5°, respectively. To further reduce the effects of confounding factors, including signals from white matter and cerebrospinal fluid, the mean time series of all voxels across the whole brain were removed from the data via linear regression. The resulting maps were spatially normalized into a standard stereotaxic space at a resolution of 3 mm × 3 mm × 3 mm using an echo-planar imaging template. After normalization, the images were smoothed using the full width at half maximum of the Gaussian kernel of 6 mm to decrease spatial noise.

### Seed Selection Using mALFF and FC Analyses

**FIGURE 1:**
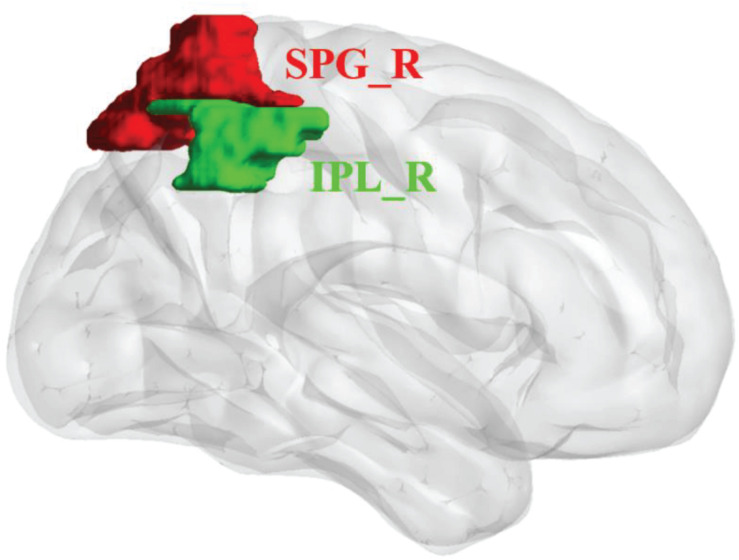
Regions of interest (ROIs) for network analysis of rs-fMRI data of upper-limb amputees (ULA) and healthy control (HC) subjects. Three-dimensional rendering showing the approximate locations of ROIs (colored patches) in parietal cortex. SPG_R, right superior parietal gyrus (red); IPL_R, right inferior parietal lobule (green).

We analyzed the mean amplitude of low-frequency fluctuations (mALFF) and FC to define body image disturbance-related brain regions in patients with upper-extremity amputations. We selected two cortical areas from a freely available atlas of regions defined by correlated activation patterns (Shirer et al., 2012). These regions of interest (ROIs) were the right superior parietal gyrus (SPG_R) and right inferior parietal lobule (IPL_R). Two ROIs of cortical regions were visualized with BrainNet Viewer (Xia et al., 2013; Figure 1).

Before calculating the amplitude of low-frequency fluctuations (ALFF) value in our participants’ ROIs, all subject-level data were preprocessed to remove any signal variations and noise using the detrend and nuisance covariate regression features of the Resting-State fMRI Data Analysis Toolkit plus V1.2 (RESTplus V1.2^2^ (Song et al., 2011). The data were detrended to reduce low-frequency drift. Linear regression of the global mean signal, head motion parameters, cerebrospinal fluid signal, and white matter signal was performed to remove the effects of nuisance covariates. We calculated the mALFF value of all the subjects’ ROIs (SPG_R and IPL_R). Next, we extracted the mALFF value of each subject’s ROIs and correlated it with the subject’s clinical scale scores.

Before calculating ROIs and whole-brain FC, all subject-level data were preprocessed with detrend, nuisance covariate regression, and band-pass filter. The data were detrended to reduce low-frequency drift. Linear regression of global mean signal, head motion parameters, cerebrospinal fluid signal, and white matter signal was executed to remove the effects of nuisance covariates. The data were processed with a temporal band-pass filter (0.01–0.08 Hz) to reduce low-frequency drift and high-frequency physiological noise. Then, FC analysis was performed using the Resting-State fMRI Data Analysis Toolkit plus V1.2 (Song et al., 2011). Based on the literature and the ALFF results, SPG_R and IPL_R were defined as the FC ROI. Next, we performed seed-based whole-brain voxel-wise FC analysis by computing the temporal correlation between the mean time series of the ROIs and the time series of each voxel within the brain. Pearson correlation coefficient maps were created for each individual subject, and these were converted to *z*-values using the Fisher *z* transformation.

### Statistical Analysis

#### Analysis of Demographic and Clinical Characteristics

After validating the normality assumption, two-tailed two sample *t*-tests and chi-square tests (only for sex) were performed to compare the demographic and clinical data from the two groups (SPSS 24.0; SPSS, Inc., Chicago, IL, United States). Significance level was set at *p* < 0.05.

#### Analyses of ROIs mALFF

Independent sample *t*-tests were used for comparing differences of mALFF between ULA and HC groups. Significance thresholds for *t*-tests were set at *p* < 0.05; thresholds were corrected with the AlphaSim module of Analysis of Functional NeuroImages (AFNI) software^3^ (Cox, 1996). The results were viewed with bspmview, a graphical user interface for overlaying, thresholding, and visualizing 3D statistical neuroimages in MATLAB. The specific anatomical location of the brain regions with statistical significance in the Montreal Neurological Institute (MNI) atlas template was also determined in bspmview. mALFF values were represented by *t*-values: *t* > 0 indicated increased functional activity and *t* < 0 indicated decreased functional activity.

#### Relationships Between mALFF and Clinical Characteristics

To quantify the relationship between mALFF values of ROIs and clinical variables, correlational analyses were performed between the mALFF values of ROIs and RLP, PLP, PLS, BDI, and BAI scores in ULAs. A two-tailed partial correlation analysis was used after controlling for age, sex, educational level as confounding variables and used multiple comparisons to correct *p*-values (*p* < 0.05).

#### Analyses of FC

For seed-based whole-brain voxel-wise connectivity, two-tailed two-sample *t*-tests were performed to evaluate group-related differences between the ULAs and the HCs. Significance threshold for *t*-tests was set at *p* < 0.01 (AlphaSim corrected). The covariates of age, sex, and educational level were controlled. These statistical analyses were carried out with the SPM12 toolbox.^4^

## Results

### Demographic and Clinical Characteristics

Table 2 shows the demographic and clinical characteristics of ULA and HC participants. No significant group differences were observed regarding age, sex, or education (*p* > 0.05).

**TABLE 2:** Statistical comparison of participants’ demographic and clinical characteristics.

**Characteristic**	**ULA (*n* = 24)**	**HC (*n* = 24)**	***t*-Value or χ2**	***p*-Value**
Age (mean ± SD)	44.67 ± 8.33	44.88 ± 12.03	−0.07	0.945
Education (year; mean ± SD)	8.58 ± 4.52	8.04 ± 6.05	0.352	0.727
Sex (male/female)	5/19	11/13	3.375	0.066
Elapsed time since amputation (month; mean ± SD)	92.79 ± 123.34	–		–
Age at amputation (year; mean ± SD)	37.13 ± 10.18	–		–
Side of amputation left/right (no.)	9/15	–		–
Amputation above/below elbows (no.)	13/11	–		–

*ULA, upper-limb amputees; HC, healthy controls.*

### ROIs mALFF

**FIGURE 2:**
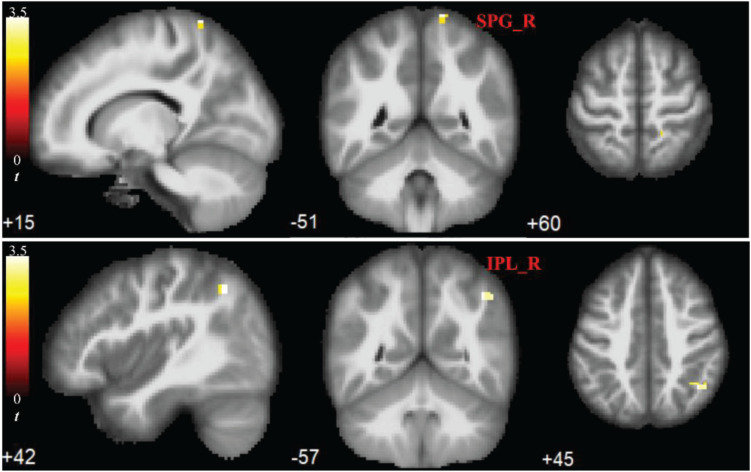
Significant increases in rs-fMRI activity of right parietal lobe months after upper-limb amputation. ROIs showing significant differences in mean amplitude of low-frequency fluctuations (mALFFs) in SPG_R and IPL_R (ULA vs. HC subjects; *p* < 0.05; AlphaSim corrected cluster). MRI slices of patients in transverse, frontal, and axial planes with color-coded *t*-values overlaid. Color-coded scale indicates increasing positive *t*-values toward yellow. SPG_R, right superior parietal gyrus; IPL_R, right inferior parietal lobule; ULA, upper-limb amputee; HC, healthy control.

Compared to the HC group, the ULA group showed a significant increase in mALFF values of both the SPG_R and IPL_R (*p* < 0.05; AlphaSim corrected cluster; see Figure 2).

### Correlations Between ROIs mALFF and Clinical Characteristics

**FIGURE 3:**
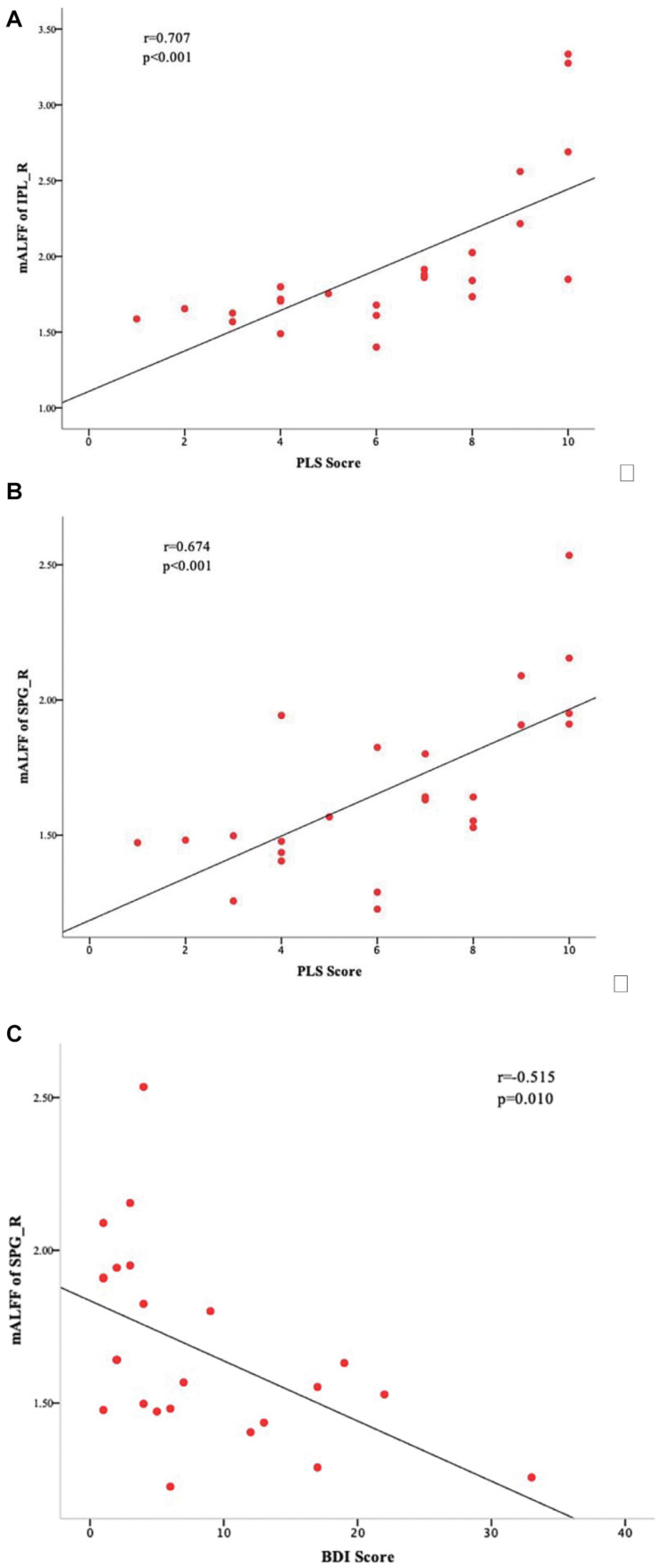
Sensory and emotional disturbances after upper-limb amputation. Correlations between ULA participants’ mALFF values in the ROIs and their clinical characteristics. **(A)** Scatter plot and statistically significant linear relationship between mALFF values of IPL_R and PLS scores (*r* = 0.707; *p* < 0.001). **(B)** Scatter plot and statistically significant linear relationship between mALFF values of SPG_R and PLS scores (*r* = 0.674; *p* < 0.001). **(C)** Scatter plot and statistically significant linear relationship between mALFF values of SPG_R and BDI score (*r* = −0.515, *p* = 0.01 < 0.05). SPG_R, right superior parietal gyrus; IPL_R, right inferior parietal lobule; PLS: phantom limb sensation; ULA, upper-limb amputee.

Correlational analyses identified a significant positive correlation between the mALFF values of IPL_R and PLS scores of the ULA group (*r* = 0.707, *p* < 0.001; Figure 3A), and a significant positive correlation between the mALFF values of SPG_R and PLS scores of the ULA group (*r* = 0.674, *p* < 0.001; Figure 3B). The analyses also identified a significant negative correlation between the mALFF values of SPG_R and BDI scores of the ULA group (*r* = −0.515, *p* = 0.01 < 0.05; Figure 3C).

### Seed-Based Whole-Brain FC

**FIGURE 4:**
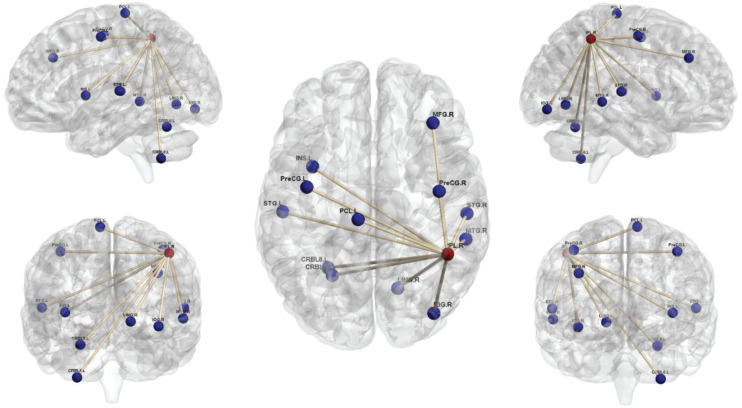
Seed-based functional connectivity maps of right inferior parietal lobe (IPL_R) in ULAs based on rs-fMRI. Compared to HC subjects, ULA subjects showed decreased functional connectivity between the seed region in the IPL_R and various brain regions (*p* < 0.01; AlphaSim corrected; voxels > 100). ULA, upper-limb amputee; HC, healthy control.

**FIGURE 5:**
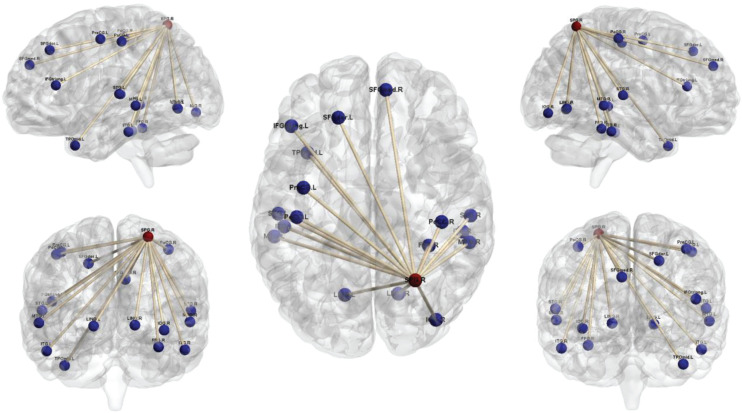
Seed-based functional connectivity maps of right superior parietal lobe (SPG_R) in ULAs based on rs-fMRI. Compared to HC subjects, ULA subjects showed decreased functional connectivity between the seed region in the SPG_R and various brain regions (*p* < 0.01; AlphaSim corrected; voxels > 100). ULA, upper-limb amputee; HC, healthy control.

Comparison of the brain connectivity maps of ULAs and HCs revealed significantly decreased FC between the IPL_R seed and many brain regions in the ULA group (*p* < 0.01; AlphaSim corrected cluster; see Table 3 and Figure 4). In the ULA group, we also observed decreased FC between the SPG_R and many brain regions (*p* < 0.01; AlphaSim corrected cluster; see Table 4 and Figure 5).

**TABLE 3:** Decreased functional connectivity in ULA participants with seed region in the IPL_R*.

**Brain region**	**Cluster size (voxels)**	**Peak coordinates (*x*/*y*/*z*; MNI)**			***t*-Value**
Temporal_Mid_R	238	57	−39	−3	−4.370
Temporal_Sup_R		45	−15	−6	−2.899
Cerebelum_L	102	−27	−63	−57	−4.354
Precentral_L	282	−33	−15	60	−3.961
Paracentral_Lobule_L		−18	−27	69	−3.113
Insula_L	430	−42	12	−9	−3.957
Temporal_Sup_L		−60	−15	12	−3.624
Lingual_R	157	21	−93	−12	−3.826
Occipital_Inf_R		42	−93	−6	−3.758
Cerebelum_L	154	−24	−78	−21	−3.493
Precentral_R	176	57	−6	48	−3.753
Frontal_Mid_R		45	12	54	−3.387

*Compared with functional connectivity of HC group (*p* < 0.01; AlphaSim corrected; voxels > 100); MNI, Montreal Neurological Institute coordinate system.

**TABLE 4:** Decreased functional connectivity in ULA participants with seed region in the SPG_R*.

**Brain region**	**Cluster size (voxels)**	**Peak coordinates (*x*/*y*/*z*; MNI)**			***t*-Value**
Temporal_Inf_L	1152	−45	−30	−27	−4.963
Frontal_Inf_Tri_L		−57	24	9	−4.794
Temporal_Pole_Mid_L		−48	12	−30	−4.751
Temporal_Sup_L	358	−54	−42	15	−4.670
Temporal_Mid_L		−63	−42	−6	−3.747
Frontal_Sup_L	598	−27	−9	69	−4.669
Postcentral_L		−45	−33	63	−4.217
Precentral_L		−45	−12	60	−4.048
Lingual_R	571	27	−72	0	−4.363
Fusiform_R		27	−51	−15	−3.825
Occipital_Inf_R		36	−87	−15	−3.619
Lingual_L	464	−9	−90	−18	−4.258
Postcentral_R	180	69	−12	27	−4.016
Temporal_Mid_R	308	63	−45	6	−4.004
Temporal_Inf_R		69	−39	−21	−3.619
Temporal_Sup_R		48	−54	21	−3.451
Frontal_Sup_Medial_R	111	12	51	45	−3.722

*Compared with functional connectivity of HC group (*p* < 0.01; AlphaSim corrected; voxels > 100); MNI, Montreal Neurological Institute coordinate system.

## Discussion

PLP and PLS are essentially illusions, misinterpreted perceptions that the limb is still present after it has been amputated. Approximately 41–76% of limb amputees report persistent PLP and PLS (Ahmed et al., 2017). This condition can be considered to be a type of body image disorder, since a coherent body image is absent (Casale et al., 2009). Due to the loss of afferent and efferent nerves in amputees, extensive cortical remodeling occurs. This plasticity represents one of the key mechanisms that lead to PLP and PLS (Makin and Flor, 2020).

The reorganization in the cerebral cortex after limb amputation is sensorimotor in nature (MacIver et al., 2008) and is believed to originate from post-amputation changes in callosal connections (Giummarra et al., 2007) or from “unmasking” of latent brain circuits that arise from permanent changes in synaptic structure (Ramachandran and Rogers-Ramachandran, 2000). Although many ULAs report PLP and PLS, non-painful phantom sensation that is related to cortical reorganization in amputees is controversial (Flor et al., 2006). The aim of the present study was to determine whether right parietal lobe plasticity following upper-limb amputation is related to aspects of PLS and body image.

Traditionally, body image disorders have been associated with damage to the convexity of the right parietal lobe posterior to the post-central gyrus (area S1) (Roth, 1949). The posterior parietal lobe is divided into the superior posterior gyrus (SPG) and inferior posterior lobules (area IPL) (Berlucchi and Vallar, 2018). Since the right SPG receives inputs from the dorsal visual stream (S1 and S2), the premotor cortex, and M1 (Van Essen et al., 2019), it seemed reasonable that the right SPG is strategically positioned to integrate disparate sensory inputs to construct a dynamic body image (Felleman and Van Essen, 1991). Therefore, we speculate that the disruption in body image after upper-limb amputation might be related to right parietal lobe remodeling.

Our rs-fMRI results revealed that the mALFF values of the ULA group were increased in both the SPG_R and IPL_R regions compared to those of the HC group. ALFF represents the intensity of local brain activity. Thus, we conclude that right parietal lobe activity increases after upper-limb amputation. This finding is consistent with related research (Flor et al., 2000; Foell et al., 2014). Increased activity in the right parietal lobe may represent functional compensation related to limb deficiencies.

Xenomelia is another kind of body image disorder that is often accompanied by the patient’s desire to self-amputate a healthy limb (McGeoch et al., 2011). Several studies confirm that reduced function of the right parietal lobe underlies this type of mental illness (McGeoch et al., 2011; Hilti et al., 2013). It is interesting that these two conditions – xenomelia and PLS – may be accounted for by opposite processes: A functional decrease in the right parietal lobe is correlated with a xenomelia patient’s desire to amputate a healthy limb, whereas a functional increase in the right parietal lobe is correlated with an amputee’s PLS. Both conditions are body disorders. Our present correlation analyses revealed a significantly positive correlation between mALFF values in IPL_R and SPG_R with PLS scores in the ULA group. We can infer that there may be a certain relationship between abnormal activation of the right parietal lobe and PLS. Thus, the function of the right parietal lobe after upper-limb amputation appears to dramatically change, affecting the quality of life of amputees.

Two different brain regions that are anatomically connected are more likely to be functionally connected (Smitha et al., 2017). With the assistance of linear temporal correlation, FC analyses using rs-fMRI data can establish that two spatially separate ROIs are connected functionally. These kind of data make it possible to analyze and understand the occurrence and development of a brain disorder to some extent (Park et al., 2018).

Through seed-based whole-brain FC calculations we found that IPL_R and SPG_R regions in ULAs exhibit decreased functional connections with many areas throughout the brain, including lingual area, precentral area, frontal area, insula area, among others (Figures 4, 5). Related studies on post-amputation changes corroborate our finding that remodeling of brain function is not limited only to local sensorimotor areas in the brain that represent the respective limb (Makin et al., 2015; Zhang et al., 2018; Molina-Rueda et al., 2019). How might these widespread changes be manifest behaviorally?

Amputee patients with a body image disorder often have reduced sensorimotor function, emotional disorders, and compromised social skills (Ko et al., 2015). The present study showed that the occipital lobe, which is involved in the expression of body image disorders, has varying degrees of reduced FC with brain areas that control sensorimotor functions and brain areas that are involved in depression and social emotions. Thus, upper-limb amputation is more than simply removal of a limb. When we did correlation analyses, we found that there are significant negative correlations between the mALFF values of SPG_R and BDI scores in amputees (see Figure 3C). We speculate that the depression scores may be related to a reduction in functional connections between SPG_R and frontal areas, because the dorsolateral superior frontal gyrus is the most important brain area associated with depression. These findings have important implications for rehabilitation. When aiming to improve remodeling of local brain areas related to the missing limb representation, it is also important to consider how to maintain FC of more remote brain areas involved in other cognitive and emotional functions affected indirectly by the amputation.

Our study has certain limitations that should be considered when interpreting the results. First, there was a limited number of subjects due to difficulty in recruiting ULAs. Of course, in future studies, more efforts will be made to recruit more upper limb amputees, so that the time and length of amputation can be effectively and reliably controlled. Second, selection bias may be present, because amputees self-selected to participate, meaning that this cooperation may relate to higher social adaptability. Third, physiological noise, such as respiratory and heartbeat fluctuations, may have influenced the stability of the rs-fMRI signals during scanning. However, we have no reason to believe that this variable would be systematically distributed to either the experimental or control group. Taken together, these limitations mean that large-scale longitudinal studies are needed for studying resting-state brain function in brain regions of patients with upper-limb amputation.

## Conclusion

Our investigation of the functional organization of parietal lobes in ULAs suggests that post-amputation reorganization is a complex phenomenon that includes functional reorganization in local areas of the respective cortical limb representations and in the degree of FC across wide areas of the brain. With further research, these results provide a reference for directing postoperative rehabilitation of not only upper-limb amputation patients but also perhaps a “road map” for investigations of other types of amputation.

## Data Availability Statement

The raw data supporting the conclusions of this article will be made available by the authors, without undue reservation.

## Ethics Statement

The studies involving human participants were reviewed and approved by Ethics Committee of Affiliated Sixth People’s Hospital of Shanghai Jiao Tong University of China (approval no. 2017-034). The patients/participants provided their written informed consent to participate in this study.

## Author Contributions

XZ was responsible for study design and manuscript revision. BB and HW were responsible for data collection and analysis. BB was responsible for manuscript writing. All authors critically reviewed the content of the manuscript and read and approved the final manuscript.

## Conflict of Interest

The authors declare that the research was conducted in the absence of any commercial or financial relationships that could be construed as a potential conflict of interest.

## Footnotes

1 http://www.fil.ion.ucl.ac.uk/spm

2 http://restfmri.net/forum/rest_v12

3 https://afni.nimh.nih.gov/

4 https://www.fil.ion.ucl.ac.uk/spm/
